# The SDGs and fossil fuel subsidy reform

**DOI:** 10.1007/s10784-023-09601-1

**Published:** 2023-05-12

**Authors:** Harro van Asselt

**Affiliations:** 1 Centre for Climate Change, Energy and Environmental Law, University of Eastern Finland Law School, Yliopistokatu 2, FI-80101 Joensuu, Finland; 2 Copernicus Institute of Sustainable Development, Princetonlaan 8a, 3584 CB Utrecht, The Netherlands

**Keywords:** Fossil fuel subsidies, International commitments, Sustainable development, Sustainable development goals, United Nations

## Abstract

This short perspective asks what is the role—and added value—of the Sustainable Development Goals (SDGs) and their associated institutional structures in the international governance of fossil fuel subsidies and their reform? It argues that whilst some progress has been made, notably through developing a methodology to define and measure fossil fuel subsidies, countries have only to a very limited extent followed up through indicator reporting and through their Voluntary National Reviews. Nevertheless, the SDGs can help highlight the various sustainable development dimensions of fossil fuel subsidies and support ongoing efforts to strengthen transparency, thereby indirectly helping to drive reform at the national level.

## Introduction: the persistent problem of fossil fuel subsidies

The case against fossil fuel subsidies is getting stronger and stronger, with evidence pointing to their adverse environmental, economic, and social impacts. Environmentally, they contribute to climate change and lead to harm to public health through air pollution. Economically, they pose a significant fiscal cost and divert scarce public resources away from other development priorities. And socially, they disproportionately benefit richer parts of the population (Couharde & Mouhoud, 2020; IPCC, 2023; Skovgaard & van Asselt, 2019). In short, fossil fuel subsidy reform can help advance sustainable development across all its dimensions.

However, not only do fossil fuel subsidies persist, they have reached new heights, particularly in the wake of the COVID-19 pandemic. The latest estimate by the International Energy Agency (IEA) of subsidies for the consumption of fossil fuels topped US$1 trillion (IEA, 2023), not even counting subsidies for fossil fuel production. The problem therefore remains in urgent need of addressing.

Since 2009, when the Group of 20 (G20) agreed to “phase out and rationalise over the medium-term inefficient fossil fuel subsidies whilst providing targeted support for the poorest” (G20, 2009), states have adopted various soft international commitments to reform fossil fuel subsidies. Amongst these is Sustainable Development Goal (SDG) Target 12.c, which marks the first time that all countries in the world—not just a subset of countries such as the G20—made a commitment to reform fossil fuel subsidies. Specifically, countries agreed to:

Rationalise inefficient fossil fuel subsidies that encourage wasteful consumption by removing market distortions, in accordance with national circumstances, including by restructuring taxation and phasing out those harmful subsidies, where they exist, to reflect their environmental impacts, taking fully into account the specific needs and conditions of developing countries and minimising the possible adverse impacts on their development in a manner that protects the poor and the affected communities (UN, 2015, 23).

Fossil fuel subsidies are also indirectly targeted through SDG 14.6—which targets fisheries subsidies—given that 22% of fisheries subsidies consist of fuel subsidies (Sumaila et al., 2019).

Since the adoption of the G20 commitment, a variety of international institutions has become involved in addressing fossil fuel subsidies (van Asselt & Verkuijl, 2021). These include international economic institutions such as the World Bank, Organisation of Economic Co-operation and Development (OECD), and the International Monetary Fund (IMF) (Skovgaard, 2021). Fossil fuel subsidies are also beginning to be addressed through trade agreements, and a group of countries has indicated their intention to develop rules on the issue through the World Trade Organisation (Asmelash, 2022), following the successful conclusion of an Agreement on Fisheries Subsidies in 2022. In addition, the United Nations climate regime has identified fossil fuel subsidy reform as a climate change mitigation measure (UNFCCC, 2022, para. 36).

This raises the question: what is the role—and added value—of the SDGs and their associated institutional structures in the international governance of fossil fuel subsidies and their reform? In this short perspective, I argue that whilst some progress has been made, notably through developing a methodology to define and measure fossil fuel subsidies, countries have only to a very limited extent followed up. Nevertheless, the SDGs can help to underscore the various sustainable development dimensions of fossil fuel subsidies, and support ongoing efforts to strengthen transparency, thereby helping to drive national-level reform.

## The (limited) effects of SDG 12.c

### Data collection efforts

Perhaps unsurprisingly, Target 12.c includes vague language (“rationalise”, “inefficient”, “encourage wasteful consumption”) and flexibilities (“in accordance with national circumstances”) that offer countries significant room for manoeuvre (van Asselt & Skovgaard, 2021). Whilst Target 12.c is thus a rather “soft” commitment in terms of its specificity and level of obligation (cf. Abbott et al., 2000), the SDGs importantly also provide for a follow-up and review process, which in theory holds potential to strengthen transparency and accountability. As part of this process, a specific indicator for Target 12.c was agreed in 2017, namely the “[a]mount of fossil fuel subsidies per unit of GDP (production and consumption) and as a proportion of total national expenditure on fossil fuels” (Indicator 12.c.1). Under the auspices of the United Nations Environment Programme (UNEP), the custodian UN agency responsible for collecting data on Target 12.c (including country-level data), a methodology for defining and measuring fossil fuel subsidies was developed (UNEP, 2019). Applying this methodology, national statistical offices and other government departments can monitor and report on fossil fuel subsidies, and thereby measure progress towards the achievement of Target 12.c.

As custodian agency, UNEP publishes data on fossil fuel subsidies—including total amounts of subsidies, subsidies as a proportion of GDP, and subsidies per capita (UN Department of Economic and Social Affairs, n.d.). In most cases, however, the published data comes from previously existing sources, namely the fossil fuel subsidy data sets maintained by three international organisations: the IEA, OECD, and IMF. In only a few cases—Armenia, Ireland, Luxembourg, Poland, Singapore, Slovakia, and the United Kingdom—does the information come directly from national statistical offices. For some countries, no data are presented at all, although these are by and large small countries (e.g., Kiribati, Malta) that are likely to have negligible or no fossil fuel subsidies. Activating national statistical offices to engage in data collection can help overcome a persistent problem related to fossil fuel subsidies, namely the lack of transparency. Moreover, data coming from national statistical offices and other government agencies, as opposed to international organisations, can also be authoritative within a country itself. Using such data, advocates for reform at the domestic level can strengthen their case. For SDG 12.c to have an impact beyond existing data collection efforts, it will therefore be important for national statistics offices to internalise and apply the methodology for Indicator 12.c.1. Given that national statistical offices have struggled to collect data related to the SDG’s environment-related indicators (Campbell et al., 2020), it is encouraging that there are ongoing efforts by UNEP and other organisations to strengthen capacity amongst statistical offices to collect data and report on Indicator 12.c.1, one of the environment-related indicators (Green Fiscal Policy Network, 2021). Such capacity-building efforts should ideally focus on countries where no data are being collected systematically and/or countries that according to OECD and IEA data have significant levels of subsidies.

### Voluntary national reviews

Another way in which the SDGs could add value is through countries’ Voluntary National Reviews (VNRs), which countries submit to report on progress towards the SDGs. An analysis of the VNRs submitted to date yields several initial insights^1^:

- Even though all G20 countries made a commitment to phase out and rationalise fossil fuel subsidies in 2009, only one country (Indonesia in 2021) explicitly reports on progress made with regard to Target 12.c. One country (United Kingdom in 2019) claims not to have any subsidies whatsoever, whereas another country (Germany in 2021) mentions fossil fuel subsidies in the context of SDG 7 (on affordable and clean energy).
- Even some countries that have been advocating for fossil fuel subsidy reform internationally—notably members of the “Friends of Fossil Fuel Subsidy Reform” (e.g., Ethiopia in 2022; the Netherlands in 2022)—fail to mention relevant data or measures adopted. Some of these countries (e.g., Denmark in 2021; New Zealand in 2020; Switzerland in 2022) mention their membership of this coalition in their discussion on Target 12.c.
- Nevertheless, some countries provide information on specific indicators (e.g., Angola in 2021, referring to IMF data; Bangladesh in 2020; Switzerland in 2022, reporting on “petroleum tax relief”; and Vietnam in 2018). Moreover, some countries that have undertaken reforms, such as Egypt (in its 2018 VNR, though not in its subsequent one) and Morocco (in its 2020 VNR) report on their efforts. One other country (Finland in 2020) notes that whilst it has championed fossil fuel subsidy reform internationally, it still has to put in place a plan to phase out fossil fuel subsidies at home.

In short, with some exceptions, it seems that VNRs are not used by countries to report information about the level of fossil fuel subsidies, the reform efforts undertaken, and/or the success of these efforts. This may be due to various reasons. First and foremost, for many countries, data constraints are likely to still play a major role. Although these constraints could be overcome by referring to data collected by international organisations, non-members of the OECD and IEA may not wish to defer to data collected by these organisations. Second, and related to this, the methodology for defining and measuring fossil fuel subsidies was only finalised in 2019 (with some data only being collected from 2020 onwards; see UN Statistics Division, 2021), and training activities are still ongoing, meaning that some countries may not yet have had a chance to reflect the information in their latest VNR. Third, some countries have already used other processes to report on fossil fuel subsidies and reform efforts, and may not see the need to disclose this information yet again. For instance, several G20 and APEC (Asia–Pacific Economic Co-operation) members have already undergone voluntary peer reviews of their fossil fuel subsidies (OECD & IEA, 2021). Lastly, perhaps a less charitable—though not less plausible—explanation is that some countries may simply be unwilling to report on fossil fuel subsidies to avoid scrutiny.

## Conclusion: Can the SDGs advance fossil fuel subsidy reform?

With SDG 12.c, another international process has been created that seeks to promote fossil fuel subsidy reform. Some progress has been made, notably agreement on a methodology for defining and measuring fossil fuel subsidies, which could help to strengthen national capacities and harmonise monitoring and reporting of subsidies. However, the lack of follow-up by countries—either through data collection efforts or through their VNRs—is concerning. The limited activity by countries on fossil fuel subsidies in the context of the SDGs follow-up and review process stands in sharp contrast with another output of the SDGs process, the Global Sustainable Development Report, which includes a call to “[p]hase out direct and indirect fossil fuel subsidies by 2025 in developed countries and by 2030 in developing countries” (Independent Group of Scientists appointed by the Secretary-General, 2019).

Whilst this may be a goal worth striving for (even though time is quickly running out), the question remains whether the SDGs can play an instrumental role in achieving this aspiration. Arguably, the broad scope of the SDGs makes the institutional process overseen by the High-Level Political Forum (HLPF) a less-than-ideal venue to address the politically vexed question of fossil fuel subsidy reform. At the same time, it is precisely this holistic nature of the SDGs—spanning environmental, economic, and social dimensions—that make the SDGs process highly suitable for addressing fossil fuel subsidies. In this regard, there may still be ways in which the SDGs process can add value.

One of these ways was already hinted at above, and that is through the training of government officials. Building on experiences by organisations such as the OECD, which receives information about fossil fuel subsidies from its members, international agencies such as UNEP and the OECD can not only help government officials in identifying and measuring subsidies, but also help bring about some level of harmonisation in a field characterised by definitional uncertainty (Garrett et al., 2021; Koplow, 2018). Second, the HLPF is mandated not only to oversee the follow-up and review process of the SDGs, but also to “facilitate sharing of experiences, including successes, challenges and lessons learned, and provide political leadership, guidance and recommendations for follow-up” (UN, 2015, 34). Whilst the HLPF’s performance in terms of providing political leadership and guidance has been disappointing thus far (Hege et al., 2020), advocates of fossil fuel subsidy reform could seek to ensure that messages like the one from the 2019 Global Sustainable Development Report are followed up by actionable recommendations in ministerial declarations adopted at the end of HLPF sessions, particularly in years in which SDG 12 is one of the focal areas. However, this possibility may be limited by the consensus-based decision-making process at the HLPF, which has meant that ministerial declarations do not go much beyond the status quo (Beisheim & Fritzsche, 2022).

Fossil fuel subsidy reforms are driven largely by domestic economic and political considerations. Whilst expectations about the SDGs process facilitating reform efforts should therefore be tempered, the SDGs can nevertheless play a role in changing domestic politics by raising or improving awareness of the problem and shedding light on the scale of the problem. Doing so can strengthen domestic coalitions in favour of reform. Indirectly, the SDGs process can thus have an impact on fossil fuel subsidy reform.

## Footnotes

1 English-language VNRs up to March 2023 were checked for the terms “fossil fuel”/”fossil fuels”, “fuel subsidies”, and “12.c”. All VNRs are available through https://hlpf.un.org/vnrs.
